# Draft Genome Sequence of *Olsenella scatoligenes* SK9K4^T^, a Producer of 3-Methylindole (Skatole) and 4-Methylphenol (*p*-Cresol), Isolated from Pig Feces

**DOI:** 10.1128/genomeA.00042-16

**Published:** 2016-02-25

**Authors:** Xiaoqiong Li, Ole Højberg, Samantha Joan Noel, Nuria Canibe, Bent Borg Jensen

**Affiliations:** Department of Animal Science, Aarhus University, Tjele, Denmark

## Abstract

*Olsenella scatoligenes* SK9K4^T^ is a strictly anaerobic bacterium isolated from pig feces that produces the malodorous compounds 3-methylindole (skatole) and 4-methylphenol (*p*-cresol). Here, we report the 2.47 Mbp draft genome sequence of SK9K4^T^, exploring pathways for the synthesis of skatole and *p*-cresol from the amino acids tryptophan and tyrosine, respectively.

## GENOME ANNOUNCEMENT

The cytotoxic and malodorous compounds 3-methylindole (skatole) and 4-methylphenol (*p*-cresol) are produced from the anaerobic degradation of l-tryptophan and l-tyrosine, respectively (1, 2). Malodorants from food production animals are both public nuisances and health concerns. Skatole is also the main compound that causes boar taint, which is an offensive odor and flavor present in the meat of some male pigs (3). Only a few cultured microorganisms are reported to produce skatole and, up to now, only four (*Clostridium scatalogenes* [4], *C. drakei* [5], *Olsenella scatoligenes* [6], and *O. uli* [7]) have been fully characterized and made available in culture collections. Unlike *C. scatalogenes* and *C. drakei*, which produce skatole directly from tryptophan (2), *O. scatoligenes* and *O. uli* synthesize skatole by decarboxylating the intermediate indole-3-acetic acid (IAA), the proximate precursor of skatole (6). Notably, *O. scatoligenes* is the only skatole-producing bacterium isolated from the pig gut. Consequently, the genome sequence of this bacterium is of interest as it could potentially be used to elucidate the skatole metabolic pathways, hence facilitating the reduction of skatole production and, thus, boar taint in pigs.

Genomic DNA from *O. scatoligenes* SK9K4^T^ was extracted and purified as previously described (6). A DNA library with a read length of 90 bp and insert size of 500 bp was constructed and then sequenced on an Illumina HiSeq 2000 platform (Beijing Genomics Institute [BGI], Shenzhen, China), following the manufacturer’s instructions. Removal of adaptors, low-quality reads, poly-N sequences, error paired-end reads, and duplications (8) resulted in 250 Mbp of clean data. Reads were assembled into 16 contigs (>200 bp) with approximately 101× coverage using SPAdes v3.6.1 (9) and the draft genome was then annotated using Prokka v1.1 (10).

The draft genome sequence of *O. scatoligenes* has a total length of 2,469,565 bp, and a *N*_50_ length of 594,252 bp, comprising 2,111 protein-coding sequences, 3 rRNAs (including 1 5S, 1 16S, and 1 23S), and 48 tRNAs genes. Using the gene marker set for the family *Coriobacteriaceae*, CheckM v1.0.3 (11) estimated the genome to be 99.0% complete. It has a G+C content of 62.4%, consistent with our previous report (62.1 mol%) (6). As expected, the *O. scatoligenes* genome contains 4-hydroxyphenylacetate decarboxylase (4-Hpd) (EC 4.1.1.83) genes, verifying that it possesses the tyrosine degradation IV (to 4-methylphenol) pathway (12). Through comparative genomics, we have also found that *O. uli* possesses 4-Hpd genes, which has previously been reported in *Clostridia* only (13). Moreover, *O. scatoligenes* also has an aliphatic amidase (EC 3.5.1.4) gene, which is required to produce IAA from tryptophan (14). However, *O. scatoligenes* is not able to synthesize IAA (6). Since the enzymology of skatole synthesis has not been characterized so far, very little is known about the genetics of this pathway. Nevertheless, candidate genes for skatole production can potentially be identified by comparative genomics.

### Nucleotide sequence accession numbers.

The *O. scatoligenes* SK9K4^T^ genome sequence has been deposited at DDBJ/EMBL/GenBank under the accession number LOJF00000000. The version described in this paper is version LOJF01000000.
